# Enhancing Comprehensive Assessments in Chronic Heart Failure Caused by Ischemic Heart Disease: The Diagnostic Utility of Holter ECG Parameters

**DOI:** 10.3390/medicina60081315

**Published:** 2024-08-14

**Authors:** Ștefania-Teodora Duca, Ionuț Tudorancea, Mihai Ștefan Cristian Haba, Alexandru-Dan Costache, Ionela-Lăcrămioara Șerban, D. Robert Pavăl, Cătălin Loghin, Irina-Iuliana Costache-Enache

**Affiliations:** 1 Department of Internal Medicine I, Faculty of Medicine, University of Medicine and Pharmacy “Grigore T. Popa”, 700115 Iasi, Romania; mihai.haba@umfiasi.ro (M.Ș.C.H.); dan-alexandru.costache@umfiasi.ro (A.-D.C.); irina.costache@umfiasi.ro (I.-I.C.-E.); 2 Department of Cardiology, “St. Spiridon” Emergency County Hospital, 700111 Iasi, Romania; ionut.tudorancea@umfiasi.ro; 3 Department of Morpho-Functional Science II-Physiology, University of Medicine and Pharmacy “Grigore T. Popa”, 700115 Iasi, Romania; ionela.serban@umfiasi.ro; 4 Department of Cardiovascular Rehabilitation, Clinical Rehabilitation Hospital, 700661 Iasi, Romania; 5 Faculty of Health Sciences and Sport, University of Stirling, Stirling FK9 4LA, UK; d.r.paval@stir.ac.uk; 6 Department of Internal Medicine, Cardiology Division, University of Texas Health Science Center, Houston, TX 77030, USA; catalin.loghin@uth.tmc.edu

**Keywords:** ischemic heart disease, chronic heart failure, 24 h Holter ECG

## Abstract

*Background and Objectives*: Chronic heart failure (CHF) caused by ischemic heart disease (IHD) is the leading cause of death worldwide and presents significant health challenges. Effective management of IHD requires prevention, early detection, and treatment to improve patient outcomes. This study aims to expand the diagnostic utility of various 24 h Holter ECG parameters, such as T-wave alternans (TWA), late ventricular potentials (LVPs), and heart rate variability (HRV) in patients with CHF caused by IHD. Additionally, we seek to explore the association between these parameters and other comorbid conditions affecting the prognosis of CHF patients. *Materials and Methods*: We conducted a prospective case–control study with 150 patients divided into two subgroups: 100 patients with CHF caused by IHD, and 50 patients in the control group. Data included medical history, physical examination, laboratory tests, echocardiography, and 24 h Holter monitoring. *Results*: Our comparative analysis demonstrated that both TWA and LVPs were significantly higher in patients with CHF compared to the control group (*p* < 0.01), indicating increased myocardial electrical vulnerability in CHF patients. Both time and frequency-domain HRV parameters were significantly lower in the CHF group. However, the ratio of NN50 to the total count of NN intervals (PNN50) showed a borderline significance (*p* = 0.06). While the low-frequency (LF) domain was significantly lower in CHF patients, the high-frequency (HF) domain did not differ significantly between groups. Acceleration and deceleration capacities were also significantly altered in CHF patients. Categorizing CHF patients by left ventricular ejection fraction (LVEF) revealed that the mean of the 5-min normal-to-normal intervals over the complete recording (SDNN Index) was significantly higher in patients with LVEF ≥ 50% compared to those with CHF with reduced EF and CHF with mildly reduced EF (*p* < 0.001), whereas the other HRV parameters showed no significant differences among the groups. *Conclusions*: Holter ECG parameters can become a reliable tool in the assessment of patients with CHF. The integration of multiple Holter ECG parameters, such as TWA, LVPs, and HRV, can significantly enhance the diagnostic assessment of CHF caused by IHD. This comprehensive approach allows for a more nuanced understanding of the patient’s condition and potential outcomes.

## 1. Introduction

Ischemic heart disease (IHD) is the most common cardiovascular ailment globally and the leading cause of death, being a risk factor for the development of chronic heart failure (CHF). Often referred to as ischemic cardiomyopathy, HF due to IHD emerges from the progressive deterioration of myocardial function resulting from chronic ischemia and myocardial infarction [1,2,3]. Also, it necessitates a multifaceted strategy encompassing prevention, early detection, and effective treatment to enhance patient survival and health outcomes [4,5].

Research on CHF has consistently demonstrated a poor prognosis and increased mortality in patients with ischemic etiology, particularly those with heart failure with reduced ejection fraction (HFrEF, EF < 40%) [6]. Despite the availability of established therapies that enhance symptoms, quality of life, and outcomes for HFrEF, the long-term prognosis for HF patients is still concerning, with a 5-year mortality rate nearing 50% after diagnosis [7,8]. Consequently, the pursuit of strategies that improve patient prognosis and decrease mortality is a critical objective in the management of HF in order to achieve accurate mortality risk assessments [8,9].

The outlook for patients with ischemic heart disease is influenced by several factors, including the degree of coronary artery involvement, the presence of additional risk factors, and compliance with prescribed treatment regimens [6]. As such, non-invasive risk stratification has become a clinical priority to pinpoint and manage individuals at high risk [6,8]. 

The left ventricular ejection fraction (LVEF) serves as the primary metric for determining which patients with ischemic cardiomyopathy are at the highest risk of cardiac mortality and thus require urgent preventive interventions [10,11]. Additionally, the N-terminal-pro hormone B-type natriuretic peptide (NT-proBNP) test is highly valuable in the screening, diagnostic, and prognostic assessment of various HF conditions, highlighting its indispensable role in clinical practice [10,12]. 

**Figure 1:**
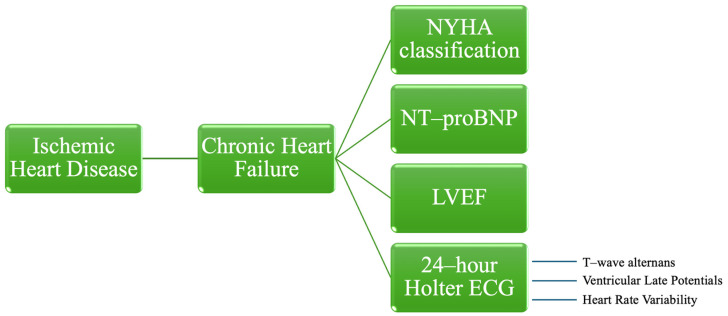
Risk stratification parameters for patients with chronic heart failure caused by ischemic heart disease. NYHA—New York Heart Association, NT-proBNP—N-terminal-pro hormone B-type natriuretic peptide, LVEF—left ventricular ejection fraction, ECG—electrocardiography.

In the literature, established predictors associated with HF, including the New York Heart Association (NYHA) classification, LVEF, and NT-proBNP are thoroughly documented. However, the evaluation of several noninvasive Holter electrocardiographic (ECG) risk stratifiers, which indicate depolarization abnormalities, repolarization abnormalities, and autonomic imbalances, remains insufficiently explored to date [11,13,14,15] (Figure 1).

Holter ECG is an indispensable tool for elucidating cardiac arrhythmias in patients with CHF. The continuous rhythm monitoring over a 24 h period facilitates the detection of atrial fibrillation, ventricular tachyarrhythmias, and other arrhythmic disturbances [16,17]. Moreover, the assessment of heart rate variability through Holter ECG provides valuable prognostic information. The integration of Holter ECG into the diagnostic and prognostic evaluation of CHF patients has significantly advanced clinical management [16,17,18].

Within hospital settings, Holter ECG monitoring proves particularly valuable in evaluating heart failure patients presenting with unexplained syncope or palpitations. Holter ECG also plays a role in monitoring the effectiveness of anti-arrhythmic treatments and in determining the optimal timing for interventions such as the implantation of cardiac devices. While in-hospital Holter ECG is indicated in certain scenarios, including post-acute coronary syndrome or in patients exhibiting hemodynamic instability, its routine use necessitates careful consideration [19,20]. It is also utilized after cardiac surgery or device implantation to monitor for arrhythmic events that may require additional intervention. However, in critically ill patients, continuous bedside monitoring is often prioritized, with telemetry or implantable loop recorders frequently being preferred over Holter ECG in these circumstances [19,21,22].

Holter ECG has demonstrated utility in identifying patients at heightened risk for sudden cardiac death, thereby informing therapeutic decision making. However, the prognostic efficacy of Holter-derived parameters, such as heart rate variability and late potentials, remains subject to ongoing investigation with inconsistent findings. The inherent limitations of Holter ECG, including the intermittent nature of arrhythmias and the potential for false-positive results, necessitate a comprehensive diagnostic approach [23,24]. The relatively brief monitoring period may preclude the detection of infrequent arrhythmic events, while patient adherence to device wearability can introduce variability in data quality [17,18]. To optimize diagnostic accuracy, Holter ECG should be integrated with other diagnostic modalities, such as echocardiography and cardiac magnetic resonance imaging [19,25,26].

Continuous Holter ECG monitoring has identified abnormalities that enhance understanding of the pathogenesis of cardiovascular death. Although extensive research has documented these Holter ECG markers, none surpass the LVEF in identifying at-risk individuals among those with reduced LVEF [14]. Prognostic data for these markers in patients with ischemic cardiomyopathy remain sparse. The effectiveness of noninvasive Holter ECG parameters, including T-wave alternans (TWA), ventricular late ventricular potentials (LVPs), and heart rate variability (HRV), in risk stratification for this patient cohort has not been fully determined [14,15,16]. Investigating the supplementary value of both resting and continuous Holter ECG alongside LVEF may improve the precise targeting of implantable cardioverter-defibrillators (ICDs) and other invasive therapies in high-risk cardiac groups [12,15,27].

Heart rate variability is an indicator of autonomic nervous system function that non-invasively measures the variations in time between consecutive heartbeats, known as RR intervals. It reflects the dynamic capacity of the heart, and the broader human physiological response, to adapt through compensatory mechanisms to fluctuating external environmental conditions [28,29]. Governed by the autonomic nervous system, which includes the sympathetic and parasympathetic branches functioning in an antagonistic relationship, HRV represents the non-stationary equilibrium maintained in the cardiovascular system [29,30]. These branches regulate visceral functions, thereby preserving vital physiological balances that manifest as variability in heart rate [31,32]. The use of HRV in clinical assessments, specifically for patient risk stratification, is advocated due to its provision of valuable prognostic information independent of other factors [32,33].

The phenomenon of T-wave alternans, which exhibit a repetitive ABABAB sequence in the amplitude and morphology of the T-wave, correlates with an elevated risk of arrhythmic episodes [34,35,36]. Recognized as a noninvasive metric, TWA is utilized to stratify the risk of sudden cardiac death (SCD) and related severe cardiac outcomes [35,37]. Through the use of Holter ECG recordings, TWA analysis serves as a pivotal alternative method for classifying patients who are at an increased risk of critical cardiac events [36,37]. 

Ventricular late potentials, as non-invasive ECG markers, have recently garnered increased attention for their role in predicting sudden cardiac death and lethal arrhythmias associated with heart failure from ischemic heart disease. Utilizing LVPs in conjunction with routine parameters, such as TWA, HRV, and non-sustained ventricular tachycardia (NSVT), has proven more effective than relying solely on LPs for risk assessment. Moreover, the synergistic use of LVPs and TWA has been shown to be beneficial in identifying patient groups at high risk, not only in the context of ischemic heart disease but also across various forms of organic heart disease [38,39].

This study was designed to assess the diagnostic utility of various Holter ECG parameters, such as TWA, LVPs, and HRV, in individuals suffering from ischemic cardiomyopathy. Previous investigations have often limited their focus to only one or two of these metrics within identical cohorts, thus constraining a comprehensive analysis. Additionally, this research aimed to determine the association between these Holter ECG parameters and other comorbid conditions known to adversely affect prognosis in patients with CHF.

## 2. Materials and Methods

### 2.1. Study Design, Patients, and Investigations

We conducted a prospective case–control study involving 100 patients who presented to St. Spiridon County Hospital between August 2022 and April 2023 with a diagnostic of ischemic cardiomyopathy. All study participants had a pre-existing diagnosis of CHF secondary to IHD, established at least one month prior to enrollment. The study population encompassed patients representing the full spectrum of heart failure, ranging from HFrEF to heart failure with preserved ejection fraction (HFpEF), inclusive of heart failure with mildly reduced ejection fraction (HFmrEF). Clinical symptoms such as dyspnea, fatigue, and ankle edema, accompanied by observable signs of heart failure were mandatory for the diagnosis of HF, as the guidelines set forth by the European Society of Cardiology (ESC) mentioned [40]. Therefore, the diagnosis was substantiated by objective evidence of cardiac dysfunction, indicated by NT-pro BNP levels of at least 125 pg/mL and supported by echocardiographic assessment.

Ischemic cardiomyopathy was classified based on the simultaneous presence of a pre-existing diagnosis of HF and confirmed atherosclerotic coronary lesions exceeding 75%, as documented through coronary arteriography in the patients’ medical records. The study population encompassed individuals with a history of IHD, characterized by a spectrum of coronary artery disease severity, from single to triple vessel involvement, irrespective of revascularization status. Additionally, participants presented with either a history of chronic coronary syndrome or a prior episode of acute coronary syndrome followed by revascularization or conservative management resulting in CHF. Aortocoronary bypass surgery was not performed in any study participant. Study participants were selected based on a history of IHD diagnosed at least one month prior to enrollment. Participants were required to exhibit no rapid or gradual onset of HF symptoms and signs, demonstrating clinical stability for a minimum of one month prior to the current evaluation through biomarkers collection and Holter ECG monitoring. These aspects were required in order to avoid instances of AHF or acute decompensation of CHF.

The control group consisted of 50 individuals with no history of heart failure or ischemic heart disease.

Exclusion criteria consisted of patients who refused to provide informed consent upon admission. The study did not include pregnant women or individuals under the age of 18. Patients receiving antineoplastic treatment or with active malignancies, and individuals with comorbid conditions associated with a life expectancy of less than one year were excluded. Moreover, we excluded the patients unable to undergo a comprehensive physical or echocardiographic examination due to factors such as severe thoracic malformations or recent thoracic surgery. Additionally, patients with thyroid disorders, acute or chronic inflammatory processes, recent major surgical procedures, or NT-proBNP levels at admission below the ESC’s recommended threshold of 125 pg/mL were excluded. Lastly, we excluded the patients with a recent history of acute coronary syndrome within 21 days before admission, those with atrial fibrillation, a documented history of sustained ventricular tachycardia, or those with cardiac implantable electronic devices. 

To ensure the reproducibility of our statistical analysis, we utilized data collected exclusively at the time of patient enrollment. Upon obtaining informed consent, we underwent a review of medical history, the physical evaluation, and laboratory investigations, including cardiac biomarkers, ECG, echocardiography, and a 24 h Holter monitoring. The relevant sociodemographic information, specific behavioral conditions, underlying medical conditions, medication regimens, and laboratory findings were noted. The confirmation of comorbidities was based on pre-existing records or were diagnosed during their hospitalization, adhering to established diagnostic criteria.

We systematically conducted the standardized laboratory tests, encompassing cardiac biomarkers, such as NT-proBNP, to D-dimers, complete blood count, renal and hepatic function, sodium, potassium, uric acid, total protein, C-reactive protein, micro-albuminuria, thyroid function markers, serum iron, ferritin, HbA1c, and glycemia. The primary objective was to rule out potential underlying causes, including anemia, infections, hepatic dysfunction, electrolyte imbalances, hypoalbuminemia, or thyroid disorders. NT-proBNP levels were measured using the PATHFAST Cardiac Biomarker Analyzer (LSI Medience Corporation, Tokyo, Japan), which employs a chemiluminescent enzyme immunoassay and the MAGTRATION^®^ method. The reference range for NT-proBNP is <15–128 pg/mL.

Utilizing the GE VividTM V7 ultrasound system (General Electric, Boston, MA, USA), each patient underwent the echocardiographic assessment, including the general morphofunctional cardiac characteristics, such as chamber dimension, pulmonary hypertension, or systolic and diastolic function. The determination LVEF followed Simpson’s method within the two-dimensional echocardiographic apical four-chamber view.

Patients underwent 24 h Holter ECG monitoring using a twelve-channel CardioScan DMS 300-3L and a digital recorder with ten wires, both produced by DM System Company Ltd., Beijing, China. Particular attention was given to TWA and HRV, including acceleration and deceleration capacities. The analysis specifically excluded patients with more than 10 atrial or ventricular ectopic beats per hour. Additionally, periods affected by noise, artifacts, premature beats, and post-extrasystolic pauses were carefully screened out and excluded from further investigation. The recorded data were analyzed manually, supported by CardioScan 12 software, part of the CardioScan Holter Analysis Software suite, developed by DM Software Inc., headquartered in Beijing, China.

Holter ECG and echocardiography evaluations were performed by two cardiologists, including one senior specialist. Additionally, the interpretation of the Holter ECG data was supervised by an electrophysiologist.

To assess TWA, the maximum observed TWA value in any channel was used. Time-domain analysis was employed, with a TWA value of 60 μV or greater being considered a positive finding.

For the HRV analysis, we excluded the records that contained more than 10% of artifacts. Both time and frequency domain analyses of HRV were automatically calculated and documented. Time-domain indices included several parameters that were previously described in the literature: the standard deviation of the averages of NN intervals in each 5-min segment throughout the recording (SDANN—normal values below 40 ms), the standard deviation of RR intervals for the entire recording (SDNN—normal values below 50 ms), the mean of the 5-min normal-to-normal intervals over the complete recording (SDNN index—normal values below 30 ms), the ratio of NN50 to the total count of NN intervals (PNN50—normal values below 0.75%), and the square root of the mean of the squares of successive differences between adjacent NN intervals (RMSSD—normal values below 15). The total count of all NN intervals divided by the height of the histogram of all NN intervals was measured on a discrete scale with bins of 7.8125 ms, being represented through the triangular index. Frequency–domain indices included the very-low-frequency (VLF) band (0.0033–0.04 Hz), low-frequency (LF) band (0.04–0.15 Hz), and high-frequency (HF) band (0.15–0.4 Hz) [41].

In relation to HRV, the determination of deceleration capacity (DC) and acceleration capacity (AC) utilized an innovative phase-rectified signal averaging method tailored for analyzing quasi-periodic oscillations within noisy and non-stationary signal data. The relative counts of deceleration and acceleration sequences, each consisting of 1 to 10 RR intervals, were classified into three risk categories, as previously detailed in the literature: low risk (values between 4.5 ms and 10 ms), intermediate risk (values from 2.5 ms to 4.49 ms), and high risk (values from 0 to 2.49 ms) [42].

For the examination of LVPs, the same device from DM System Company Ltd., Beijing, China, equipped with a 3-channel (orthogonal lead) setup and a 7-wire recorder, was employed. Adhering to a standardized protocol, CardioScan 12 software was utilized for the investigation. Each participant first underwent a 3-lead resting electrocardiogram with the Holter ECG DMS 300-4L device, recording 500 cardiac cycles typically within 12 to 15 min. The assessment of LVPs was subsequently repeated using the same device’s integrated software. The QRS waveforms were filtered bi-directionally within a frequency range of 40–250 Hz. The analysis of the filtered QRS complex for ventricular late potentials involved specific criteria: a filtered QRS complex duration (fQRS) greater than 114 milliseconds, the presence of low-amplitude signals (LAS) lasting over 38 milliseconds in the terminal portion of the QRS complex, and a root mean square (RMS) voltage within the terminal 40 milliseconds below 20 microvolts (μV). A diagnosis of LVPs was made if at least two of these three criteria were met [43,44].

The primary objective of this investigation was to assess the variability and diagnostic value of 24 h Holter ECG parameters in patients with CHF attributable to IHD, in comparison to a control group devoid of HF or IHD. Secondary objectives encompassed evaluating the correlations between modified Holter ECG parameters and additional potential factors that could impact the results.

### 2.2. Statistical Analysis

Statistical analyses were performed using version 4.3.2 of the R programming language. Initial evaluations of data distribution were conducted utilizing the Shapiro–Wilk test. Continuous variables displaying normal distribution were expressed as mean ± standard deviation (SD), whereas those not following a normal distribution were summarized descriptively via the median and interquartile range (IQR: 25–75%). Frequencies and percentages were employed to represent categorical variables. To assess group differences, the independent samples *t*-test was applied for normally distributed variables, while the Mann–Whitney U test was utilized for variables not conforming to a normal distribution. Pearson’s correlation coefficient (Pearson’s r) was used to explore correlations between parameters under parametric assumptions, whereas Spearman’s rank correlation coefficient (Spearman’s ρ) was applied for non-parametric data. A one-way ANOVA was conducted when more than two groups were compared. If the ANOVA results were statistically significant, a Games–Howell post hoc test was used to identify the pair(s) of groups that were different. Comparisons of categorical groups were executed using the chi-squared test. A *p*-value threshold of less than 0.05 (*p* < 0.05) was established to determine statistical significance.

### 2.3. Ethics

All patients provided written informed consent upon admission to participate in the research. This study was conducted in compliance with the ethical guidelines outlined in the 1975 Declaration of Helsinki, as updated in 2013. The study protocol received approval from the Ethics Committees of the University of Medicine and Pharmacy “Gr.T. Popa” (no. 185/12 May 2022) and the Emergency Clinical Hospital St. Spiridon (no.47/14 April 2022).

## 3. Results

### 3.1. Baseline Characteristics

We enrolled a total of 150 patients, divided into two subgroups: 100 patients with CHF caused by IHD, and 50 control patients without these conditions. Within the CHF subgroup, 32% were female and 68% were male. The average age of CHF patients was 68 ± 11 years, while the control group’s average age was 63 ± 12 years. Among the CHF cohort, 72 patients exhibited HFrEF, 26 displayed HFmrEF, and 2 patients presented with HFpEF.

Table 1 presents the demographic, clinical, and biological characteristics of the study participants. Our analysis revealed a significantly higher prevalence of major cardiovascular risk factors, such as advanced age and smoking, among patients with ischemic cardiomyopathy compared to the control group. However, no significant differences were found between the two groups in terms of other traditional cardiovascular risk factors, including gender, body mass index, or diabetes. Additionally, there was no difference in the incidence of chronic kidney disease, a notable comorbidity, between the groups. Notably, comprehensive blood tests indicated no presence of anemia, infection, or electrolyte imbalances, and confirmed normal liver and thyroid functions among the patients. 

While no instances of bundle branch block (BBB) were observed among the control group, the CHF cohort exhibited a spectrum of intraventricular conduction disturbances. Specifically, 12% of CHF patients presented with left bundle branch block (LBBB), 8% with right bundle branch block (RBBB), and 80% demonstrated normal QRS morphology. 

All patients diagnosed with CHF were administered beta-blockers at varying dosages: 64% received carvedilol, 27% were prescribed bisoprolol, and 9% were treated with metoprolol. None of the patients were on amiodarone, other antiarrhythmic agents, or other treatments known to have potential arrhythmic effects. Additionally, none of the participants in the control group were treated with beta-blockers or antiarrhythmic medications. 

Concerning echocardiographic parameters, there were significant differences between the groups in all measured variables, including chamber diameters and volumes, systolic and diastolic function, and estimated systolic pulmonary artery pressure (*p* < 0.001). However, it is important to note that no statistically significant differences were observed in the E/A ratio (*p* = 0.07) and right ventricular end-diastolic diameter (*p* = 0.31) (Table 2).

### 3.2. Diagnostic Performance of Late Ventricular Potentials and T-Wave Alternans in Chronic Heart Failure

Our study subsequently progressed to a detailed comparative analysis between the two groups, focusing on LVPs obtained from signal-averaged electrocardiography. Additionally, we evaluated TWA in both groups (Table 3).

**Figure 2:**
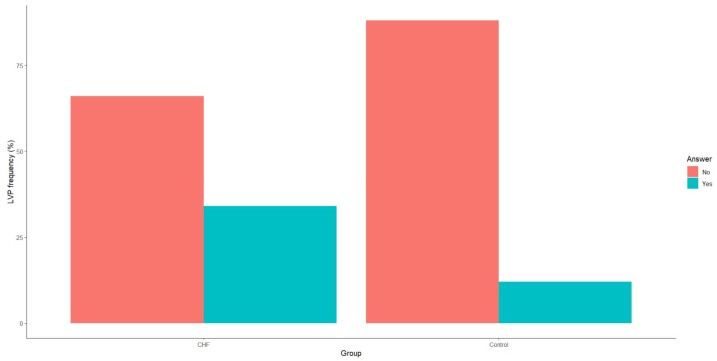
Frequency of late ventricular potentials in patients with CHF and the control group. CHF—chronic heart failure.

Both TWA and LVPs demonstrated statistically significant differences between patients with CHF and the control group, as evidenced by *p*-values < 0.01. Specifically, patients with CHF due to IHD exhibited a significantly higher incidence of LVPs (Figure 2).

**Figure 3:**
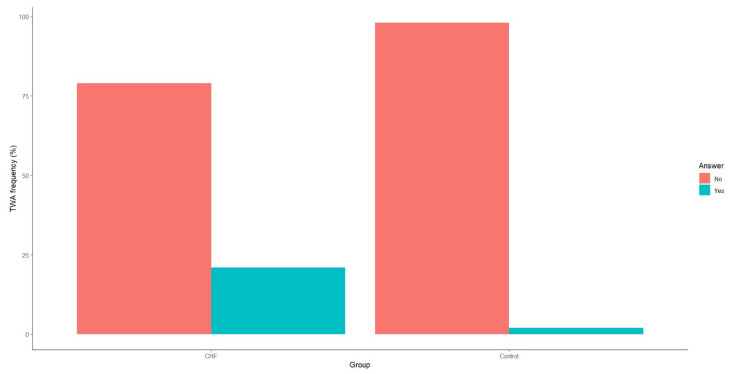
Frequency of T-wave alternans in patients with CHF and the control group. CHF—chronic heart failure.

Our analysis also revealed that TWA incidence was significantly higher in patients with CHF compared to the control group (Figure 3). 

### 3.3. Diagnostic Performance of Heart Rate Variability in Chronic Heart Failure

Our study progressed to conduct comparative assessments between the two groups, focusing on HRV parameters in both time and frequency domains. Specifically, we analyzed various HRV measures, including SDNN, SDANN, SDNN index, RMSSD, PNN50, VLF, LF, and HF components. Additionally, we evaluated acceleration and deceleration capacities (Table 4).

Our study confirmed the statistical significance of both time and frequency domain HRV parameters in patients with CHF compared to a control group. Notably, the SDNN value was significantly lower in the CHF group, with a median of 74.0 (IQR: 56.0–96.0, *p* < 0.001), and RMSSD also displayed a significant reduction, with a median of 23.0 (IQR: 16.0–34.5, *p* = 0.03). 

However, one specific time-domain parameter, PNN50, demonstrated a borderline value (median: 3.0, IQR: 0.0–8.0, *p* = 0.06) and was significantly lower in patients with CHF compared to the control group. 

Our analysis revealed a significantly lower LF value in patients with CHF compared to the control group. Specifically, the median LF value in the CHF group was 179.0 (IQR: 92.2–359.0). In contrast, the HF component did not show a statistically significant difference between the CHF patients and the control group, with a median HF value of 76.8 (IQR: 35.9–181.0, *p* = 0.11).

**Figure 4:**
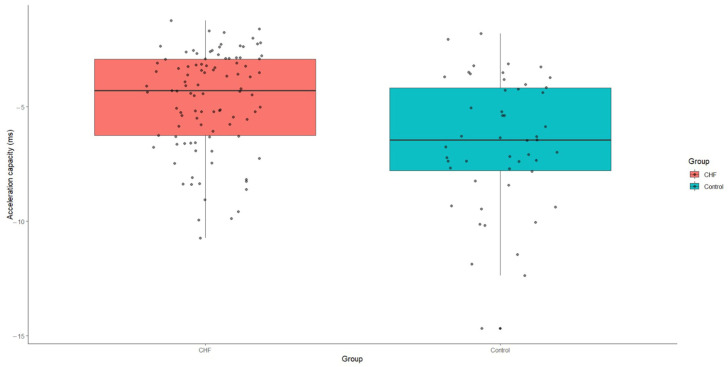
Acceleration capacity values in patients with CHF and control group. CHF—chronic heart failure.

**Figure 5:**
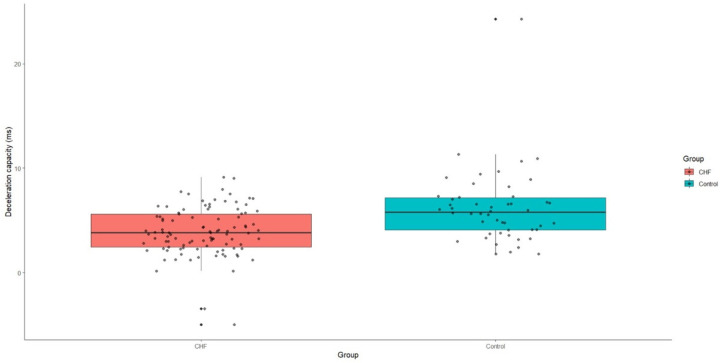
Deceleration capacity values in patients with CHF and control group. CHF—chronic heart failure.

Acceleration capacity was significantly elevated in patients with CHF relative to the control group, with a median value of −4.3 (IQR: −6.3-−2.9), *p* < 0.001. Conversely, deceleration capacity was markedly reduced in the CHF group, presenting a median value of 3.8 (IQR: 2.4–5.6), *p* < 0.001 (Figure 4 and Figure 5). 

### 3.4. Can LVEF Influence the HRV Parameters in Patients with Chronic Heart Failure?

Patients with chronic heart failure were categorized into three groups based on their LVEF: ≤40% (HFrEF), 41–49% (HFmrEF), and ≥50% (HFpEF). A comprehensive comparison of all HRV parameters, including time and frequency domains, was conducted using a one-way analysis of variance (ANOVA). 

The results indicated no significant differences among the groups for several HRV parameters: SDNN (F = 1.8, *p* = 0.32), SDANN (F = 1.8, *p* = 0.32), RMSSD (F = 3.0, *p* = 0.21), PNN50 (F = 0.8, *p* = 0.53), triangular index (F = 2.7, *p* = 0.23), VLF (F = 0.1, *p* = 0.99), LF (F = 1.3, *p* = 0.42), HF (F = 1.2, *p* = 0.43), deceleration capacity (F = 3.9, *p* = 0.16), and acceleration capacity (F = 0.9, *p* = 0.50). However, a notable exception was observed for the SDNN Index, which showed a significant difference between the groups (F = 71.8, *p* < 0.001). 

**Figure 6:**
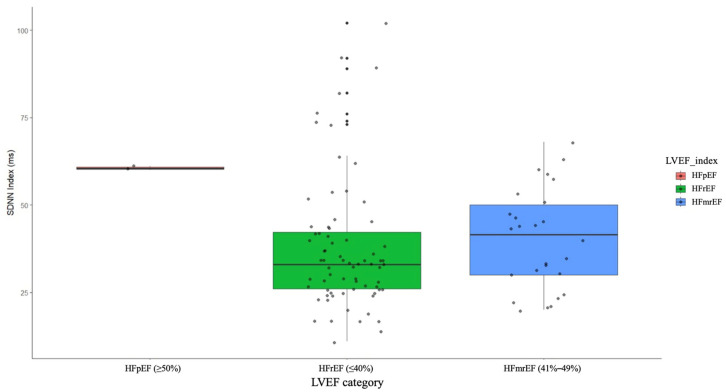
SDNN index in patients with HFrEF, HFmrEF, and HFpEF. HFrEF—heart failure with reduced ejection fraction, HFmrEF—heart failure with mildly reduced ejection fraction, HFpEF—heart failure with preserved ejection fraction.

Post hoc analysis revealed that patients with LVEF ≥50% had a significantly higher SDNN Index compared to those with HFrEF (t = −10.0, *p* < 0.001) and HFmrEF (t = −26.0, *p* < 0.001). No significant difference in the SDNN Index was found between the HFrEF and HFmrEF groups (t = −0.7, *p* = 0.79) (Figure 6).

## 4. Discussion

In IHD, chronic ischemia precipitates myocardial infarction, fibrosis, and structural remodeling of the heart muscle, resulting in diminished cardiac output [45]. The ischemic injury and subsequent ventricular remodeling compromise both systolic and diastolic functions [46]. In patients with IHD, the development of CHF involves intricate mechanisms, including neurohormonal activation, oxidative stress, and inflammatory pathways. Neurohormonal activation is pivotal in the pathophysiology of both IHD and CHF. Activation of the sympathetic nervous system is among the initial responses to reduced cardiac output, leading to the release of catecholamines. These catecholamines enhance chronotropy, inotropy, and vasoconstriction [45,46,47]. Although these effects temporarily bolster cardiac function, prolonged stimulation incurs deleterious outcomes such as heightened myocardial oxygen demand, arrhythmias, and myocyte apoptosis [46,47,48].

Despite the reliance on clinical symptoms, physical examination, laboratory investigations, and the significant contribution of imaging techniques, Holter ECG has emerged as a critical diagnostic and prognostic tool. In the management of CHF, echocardiography remains indispensable due to its non-invasive nature, widespread availability, and ability to provide real-time information [49]. However, Holter ECG monitoring is equally important in the management of ischemic cardiomyopathy, significantly contributing to risk stratification. It offers extensive diagnostic and prognostic insights into cardiac rhythm disturbances, autonomic function, and treatment efficacy, thus elucidating the complex mechanisms underlying sudden cardiac death [50].

Our study results indicated that various Holter ECG parameters, such as LVPs, TWA, and HRV, can serve as valuable diagnostic tools for patients with CHF. Additionally, these parameters can further solidify the importance of Holter ECG in managing this patient population.

Early alterations in HRV parameters in CHF typically manifest as reductions in time-domain measures such as SDNN, RMSSD, and pNN50, alongside frequency-domain measures like HF power. These initial changes are indicative of diminished parasympathetic activity and an imbalance in autonomic regulation, which are fundamental aspects of CHF pathophysiology [51]. However, in our study, we did not achieve statistical significance for pNN50 and HF power. Instead, significant findings were observed for less commonly utilized parameters, including AC, DC, and the triangular index.

The AC of HRV is a relatively novel metric that quantifies the heart’s ability to accelerate in response to physiological demands [52]. This metric is derived from advanced time-domain analysis of HRV data, reflecting the heart’s intrinsic capability to adapt to changes, particularly through the autonomic nervous system [52,53]. Conversely, the DC of HRV measures the heart’s ability to decelerate in response to physiological stimuli. DC is an important marker of autonomic nervous system function, especially vagal (parasympathetic) activity, and holds significant clinical and prognostic value. The triangular index further complements these measures by providing an overall assessment of HRV complexity and autonomic regulation in CHF patients [53,54,55].

Acceleration capacity and deceleration capacity are often more sensitive to early autonomic changes in CHF compared to pNN50 due to their specific roles in measuring distinct aspects of HRV [56]. AC, which quantifies the heart’s capacity to accelerate in response to physiological demands, is particularly adept at detecting early autonomic dysfunction because it directly measures heart rate acceleration. This metric has demonstrated clinical utility as an early indicator of autonomic imbalance, providing a more immediate reflection of autonomic changes compared to pNN50 [56,57,58]. In a similar way, DC, which assesses the heart’s ability to decelerate in response to physiological stimuli, is highly sensitive to early reductions in parasympathetic (vagal) activity. This measure captures heart rate deceleration and has been shown to be a valuable clinical marker for detecting early parasympathetic dysfunction before more generalized HRV measures like pNN50 reveal significant changes [57,59,60]. While pNN50 also reflects parasympathetic activity by quantifying the percentage of successive RR intervals differing by more than 50 ms, it is a more generalized measure of overall heart rate variability and may not capture subtle early shifts in autonomic regulation as effectively as AC and DC [60,61].

In the context of CHF, there is typically an early reduction in vagal tone coupled with a corresponding increase in sympathetic activity [60]. Both AC and DC are directly associated with these early autonomic changes and thus have the potential to reflect shifts in autonomic balance before they are detectable through broader HRV measures such as pNN50. The ability of AC and DC to identify these subtle early alterations in autonomic regulation underscores their value as diagnostic tools for the early detection and management of CHF [60,61,62]. Their application can facilitate more timely and effective therapeutic interventions, potentially improving patient outcomes through earlier and more precise management of CHF-related autonomic dysfunction. Thus, the sensitivity of AC and DC to early autonomic changes positions them as critical tools for early detection of autonomic dysregulation in CHF, offering distinct advantages over more traditional HRV measures like pNN50. Their specific and early detection of autonomic imbalances makes AC and DC indispensable in the management of CHF, paving the way for timely and effective clinical interventions [60,63,64].

Our investigation aimed to determine whether additional factors might influence HRV results. While CHF is a well-known factor affecting HRV, various other elements can also impact these parameters. Laboratory tests reveal that conditions such as thyroid dysfunction, anemia, elevated inflammatory markers, or dyselectrolytemia can alter HRV results. In our study, however, we excluded patients with these conditions to ensure a homogeneous cohort. Regarding different comorbidities, chronic kidney disease (CKD), obesity, and diabetes are known to influence HRV parameters through multiple mechanisms [65,66,67,68,69,70,71]. In diabetic patients, fluctuations in blood glucose levels, chronic inflammation, oxidative stress, insulin resistance, and metabolic syndrome contribute to heart rhythm disturbances [66,67]. CKD is characterized by the accumulation of uremic toxins, which can stimulate myocardial fibrosis, trigger inflammation, and induce oxidative stress, thereby impacting the heart’s electrical properties. Additionally, CKD can lead to fluid retention and electrolyte imbalances, particularly affecting potassium levels and HRV [68,69]. Obesity is associated with chronic low-grade inflammation, which can disrupt the autonomic nervous system and is often linked to insulin resistance and metabolic syndrome [70]. In our study, no significant differences were observed between the two groups concerning other traditional cardiovascular risk factors, including gender, CKD, body mass index, or diabetes, which could have influenced the results. However, one nonmodifiable risk factor, age, showed a statistically significant higher prevalence among older patients with CHF compared to the control group (*p* = 0.04). Age impacts HRV by causing a general decline in autonomic regulation, as evidenced by reduced time-domain and frequency-domain HRV measures and a shift towards sympathetic dominance. This reduction in HRV with aging is attributable to changes in the autonomic nervous system, cardiovascular structure and function, and overall physiological aging processes [72,73].

Arguably, the most critical factor that could influence HRV is LVEF. To explore this, patients were categorized into three groups based on their LVEF: ≤40% (HFrEF), 41–49% (HFmrEF), and ≥50% (HFpEF). Reduced LVEF is often linked with autonomic dysfunction, characterized by decreased parasympathetic activity and increased sympathetic activity, leading to lower HRV [74]. However, our study found no significant differences among these groups for most HRV parameters. The consistency of HRV across different LVEF levels can be attributed to several factors. HRV is influenced by a range of elements beyond LVEF, including autonomic nervous system activity, overall cardiovascular health, comorbid conditions, lifestyle factors, and individual physiological differences [75,76]. In some patients with reduced LVEF, compensatory mechanisms might preserve autonomic function, resulting in relatively stable HRV. These compensatory mechanisms can include neurohormonal adaptations, physical conditioning, and medical therapies [77]. A notable exception was observed for the SDNN index, which showed a significant difference between the groups. The SDNN index, reflecting overall heart rate variability and long-term cardiac dynamics, can change in patients with different LVEF values while other HRV parameters remain constant. This sensitivity to cumulative physiological and pathological processes over time allows the SDNN index to capture changes in overall cardiac function and variability that may not be detected by more specific, short-term HRV measures. Factors such as compensatory mechanisms, the impact of LVEF on cardiac output, comorbidities, and medications contribute to this phenomenon [78,79].

Regarding other Holter ECG parameters, namely TWA and LVPs, a statistically significant higher incidence was observed in patients with CHF compared to the control group. LVPs are low-amplitude signals detected at the end of the QRS complex on a high-resolution ECG, associated with delayed depolarization of the ventricles and an increased risk of arrhythmias [27,80]. TWA refers to beat-to-beat alternations in the amplitude or morphology of the T-wave on an ECG, serving as a marker of electrical instability and is linked to a higher risk of arrhythmias, particularly ventricular arrhythmias [29,81]. The elevated incidence of LVPs and TWA in patients with CHF can be attributed to several pathophysiological factors, including myocardial remodeling and fibrosis, increased electrical heterogeneity, autonomic dysfunction, ischemia and hypoxia, or ventricular dilation and hypertrophy. These factors contribute to the disruption of normal electrical conduction pathways in the heart, leading to delayed depolarization in certain ventricular regions, which are detected as LVPs on a high-resolution ECG. Additionally, the presence of TWA indicates increased electrical instability, further underscoring the arrhythmic risk in CHF patients [80,81].

Our subsequent concern was whether the severity of coronary artery disease could influence the presence and characteristics of TWA and LVPs. The severity of CAD can significantly impact LVPs and TWA through several mechanisms, such as ischemia-induced conduction delays, myocardial infarction and scar formation, myocardial remodeling and fibrosis, autonomic nervous system imbalance, electrophysiological alterations, and the development of an arrhythmogenic substrate [82]. Severe CAD disrupts normal electrical conduction pathways in the heart, leading to delayed depolarization and an increased incidence of LVPs and TWA, both of which are associated with a higher risk of ventricular arrhythmias. Chronic ischemia and infarction can induce changes in ionic currents within cardiac cells, particularly involving calcium and potassium ions. These ionic alterations can lead to beat-to-beat variability in repolarization, manifesting as TWA [82,83]. Future investigations with larger cohorts should systematically evaluate the extent of ischemic involvement and the specific anatomical localization of CAD to better understand its influence on TWA and LVPs. This approach would provide more comprehensive insights into the pathophysiological link between CAD severity and the risk of ventricular arrhythmias as indicated by these ECG parameters.

As previously outlined, all participants with CHF underwent supervised therapy for heart failure, which included beta-blockers, recognized as a primary determinant influencing HRV, as well as TWA and LVPs. Beta-blockers can significantly impact HRV, LVPs, and TWA by reducing sympathetic activity, stabilizing myocardial repolarization, exerting anti-arrhythmic effects, improving myocardial oxygen supply, and modulating calcium handling. These effects contribute to a reduction in the incidence and severity of TWA, thereby decreasing the risk of arrhythmias and enhancing overall cardiac stability [84]. Clinical evidence supports the use of beta-blockers to manage TWA and improve outcomes in patients with IHD and CHF [85]. Prospective investigations, encompassing diverse beta-blockers and their respective dosages, are imperative to ascertain not only their direct impact on Holter ECG parameters but also to elucidate potential interactions between different beta-blockers and Holter ECG outcomes. Such comprehensive exploration is essential for refining our understanding of the nuanced effects of beta-blockade on HRV, LVPs, and TWA across various patient populations and clinical contexts. Considering the wide range of beta-blockers available, including those with varying pharmacokinetic and pharmacodynamic profiles, a thorough investigation can provide valuable insights into optimizing treatment strategies for heart failure while minimizing adverse effects [86]. Future research should systematically explore potential correlations between HRV, TWA, and LVPs with specific parameters such as beta-blocker dosage and classifications. This analytical trajectory promises to provide a comprehensive understanding of the intricate interplay between beta-blocker pharmacotherapy and cardiac autonomic regulation, ultimately improving therapeutic outcomes for patients with heart failure.

While our study did not evaluate long-term adverse cardiovascular outcomes, numerous investigations have established the prognostic capacity of HRV, TWA, and LVPs for significant adverse cardiovascular events [9,12,26]. To address this limitation, we aim to incorporate data on long-term cardiovascular events and extend our follow-up in future studies.

The future perspectives for Holter ECG parameters in patients with CHF due to IHD involve advancements in several areas, aiming not only to refine risk stratification but also to guide early therapeutic interventions, thereby enhancing overall quality of life. Advanced HRV metrics and machine learning algorithms could help identify patients at high risk for adverse events, such as arrhythmias and sudden cardiac death [87,88,89]. 

Holter monitoring offers potential benefits in enhancing diagnostic and prognostic accuracy, particularly for patients presenting with syncope. A selective approach to Holter monitoring, as evidenced by recent research in syncope populations, is crucial for optimizing diagnostic yield [15]. By meticulously identifying patient subgroups most likely to benefit from this modality, clinicians can mitigate unnecessary testing while augmenting the detection of cardiac-related conditions. This tailored approach aligns with the principle of personalized medicine, enabling more efficient and effective patient evaluation. However, as highlighted by Freund et al., judicious application of Holter monitoring is imperative. Routine 24 h Holter monitoring during hospitalization should be reserved for patients with a high pre-test probability of arrhythmia-related pathology to avoid overutilization and potential adverse consequences [15,90,91].

Left ventricular ejection fraction assumes paramount significance in determining eligibility for defibrillator implantation, with LVEF values at or below the commonly accepted threshold of 35% serving as an indication for primary prevention [31]. However, risk assessment in this context is multifactorial. Future research may focus on developing more sensitive HRV-based algorithms and tools for the early detection of CHF, potentially leading to earlier intervention and improved patient outcomes. Currently, there is no universally accepted risk scoring system specifically tailored for determining defibrillator implantation suitability in heart failure patients, and none of the existing scoring systems incorporate HRV as a primary parameter [92]. The MultiSENSE study incorporated a novel HF monitoring algorithm, the HeartLogic index, developed by Boston Scientific. This algorithm, embedded within an ICD, integrates a multifaceted array of physiological data including cardiac acoustics, thoracic bioimpedance, heart rate variability, respiratory patterns, and physical activity. The study findings underscored the HeartLogic index as a robust predictor of impending HF decompensation, demonstrating its capacity for early detection [93]. The HLM score constitutes a novel paradigm for assessing CHF severity. By synthesizing cardiac, pulmonary, and systemic organ involvement, the HLM score offers a more comprehensive evaluation of disease burden compared to traditional metrics such as ejection fraction. D’Amato et al. posit that the HLM score holds significant potential as a clinical tool for risk stratification, therapeutic decision making, and patient counseling within the context of CHF management [94]. Another cornerstone in risk stratification for heart failure, the Seattle Heart Failure Model is a widely employed prognostic tool. This model integrates a comprehensive array of clinical, laboratory, and pharmacological variables to generate predictive estimates of one- and five-year mortality. Key components encompass patient demographics, physiological measures, functional status as indexed by the New York Heart Association classification, therapeutic regimens, and relevant biochemical parameters [95]. Future research should aim to develop and validate more advanced HRV metrics, including nonlinear and complex measures, to provide a more comprehensive assessment of autonomic function in CHF patients. This could significantly improve risk stratification and guide therapeutic decisions, ultimately enhancing patient care and outcomes in heart failure management.

### Limitations of the Study

The primary limitation of this study is its single-center design and relatively small patient cohort. However, the rigorous implementation of comprehensive exclusion criteria is mandatory as it facilitates an in-depth investigation of various concomitant Holter ECG parameters. Enlarging the study cohort would facilitate the conduct of more robust multivariable regression analyses, thereby enabling the development of a comprehensive risk stratification model incorporating multiple parameters. Furthermore, a larger sample size would permit a more in-depth exploration of the correlation between echocardiographic, biological, and Holter ECG findings in patients with heart failure, as well as the identification of potential moderating factors influencing these relationships. While the Holter ECG is an essential tool for monitoring cardiac activity, this study did not compare its results with those from other Holter devices. Additionally, the study population comprised patients exhibiting a range of QRS complex morphologies. While the control group demonstrated normal QRS patterns, the CHF cohort included patients with both left and right bundle branch blocks. To enhance the precision of future prognostic models, a detailed analysis differentiating these QRS patterns might provide more specific insights. Another limitation is the omission of considerations regarding the duration and severity of patients’ ischemic cardiomyopathy, which could influence the results, particularly in cases of more severe obstructive artery disease. It is important to highlight that our study cohort, primarily consisting of chronic heart failure patients, includes individuals at various stages of the disease, introducing heterogeneity that should be addressed in future research. Moreover, the limited number of patients with HFpEF may potentially influence this study’s findings. Lastly, even though all chronic heart failure patients received beta-blockers, we did not analyze potential correlations with specific beta-blocker classifications or dosages.

## 5. Conclusions

Our research aimed to expand the diagnostic applications of Holter ECG in individuals with chronic heart failure due to ischemic heart disease. Our study confirmed that most of both time and frequency-domain parameters of HRV were statistically lower in patients with CHF compared to the control group.

However, while traditional HRV parameters such as PNN50 and HF did not show statistical significance, non-traditional parameters like acceleration and deceleration capacity and the triangular index demonstrated significant diagnostic utility. These findings emphasize the importance of analyzing a comprehensive range of HRV parameters, even when traditional measures are within normal ranges. Interestingly, left ventricular ejection fraction did not influence the HRV results among CHF patients, except for the SDNN Index, suggesting a consistent degree of autonomic nervous system imbalance across all CHF patients.

Regarding myocardial electrical vulnerability, our study demonstrated a significantly higher incidence of both late ventricular potentials and T-wave alternans in CHF patients.

Therefore, integrating multiple parameters recorded through Holter ECG, such as TWA, LVPs, and HRV, can serve as a valuable diagnostic tool for diverse patient populations. Nonetheless, additional extensive multicenter studies are necessary to confirm their prognostic utility in patients with CHF due to IHD.

## Figures and Tables

**Table 1:** Baseline characteristics of patients with CHF and control group.

	Chronic Heart Failure (*n* = 100)	Control (*n* = 50)	*p*-Value
Sex (M/F)	F—32 (32%) M—68 (68%)	F—20 (40%) M—30 (60%)	0.33
Age (years)	68 ± 11	63 ± 12	0.04
Place of residence (urban/rural)	Urban—42 (42%) Rural—58 (58%)	Urban—29 (58%) Rural—21 (42%)	0.06
Smoking (pack-year)	13.2 (IQR: 0.0–27.5)	6.76 (IQR: 0.0–11.0)	0.02
Alcohol consumption (Yes/No)	Yes—16 (16%) No—84 (84%)	Yes—11 (22%) No—39 (78%)	0.37
Diabetes (Yes/No)	Yes—30 (30%) No—70 (70%)	Yes—10 (20%) No—40 (80%)	0.19
Chronic kidney disease (Yes/No)	Yes—21 (21%) No—79 (79%)	Yes—7 (14%) No—43 (86%)	0.47
Body mass index (kg/m^2^)	27.4 (IQR: 24.1–30.8)	26.9 (IQR: 23.7–32.5)	0.75
NT-proBNP (pg/mL)	2798.0 (IQR: 697.0–7589.0)	55.6 (IQR: 22.0–105.0)	<0.001
NYHA classification (I/II/III/IV)	I—6 (6%) II—50 (50%) III—40 (40%) IV—4 (4%)	N/A	N/A
Bundle branch block	LBBB—12 (12%) RBBB—8 (8%) No—80 (80%)	N/A	N/A
Beta-blockers	Carvedilol—64 (64%) Bisoprolol—27 (27%) Metoprolol—9 (9%)	N/A	N/A

CHF—chronic heart failure, M—male, F—female, NT-proBNP—N-terminal-pro hormone B-type natriuretic peptide, NYHA—New York Heart Association, LBBB—left bundle branch block, RBBB—right bundle branch block, N/A—not applicable.

**Table 2:** Echocardiographic parameters in patients with CHF and control group.

	Chronic Heart Failure (*n* = 100)	Control (*n* = 50)	*p*-Value
LVEF (%)	0.32 ± 0.10	0.55 ± 0.04	<0.001
LVEDV (mL)	195.0 (IQR: 153.0–238.0)	149.0 (IQR: 125.0–175.0)	<0.001
LVESV (mL)	129.0 (IQR: 96.0–166.0)	65.0 (IQR: 53.8–81.0)	<0.001
E/A	1.0 (IQR: 0.7–1.8)	0.9 (IQR: 0.7–1.2)	0.07
Average E/E’	11.9 (IQR: 8.1–15.6)	6.80 (IQR: 5.9–9.2)	<0.001
E/E’ lateral	9.5 (IQR: 7.1–12.6)	6.1 (IQR: 4.9–8.3)	<0.001
E/E’ septal	12.8 (IQR: 8.7–18.5)	7.6 (IQR: 6.6–10.1)	<0.001
S’ lateral (mm/s)	0.07 (IQR: 0.05–0.08)	0.09 (IQR: 0.07–0.10)	<0.001
S’ septal (mm/s)	0.06 (IQR: 0.05–0.08)	0.09 (IQR: 0.07–0.10)	<0.001
MV Dec T (ms)	178.0 (IQR: 141–215)	201.0 (IQR: 163.0–246.0)	0.03
LAVI (mL/m^2^)	22.1 (IQR: 18.4–26.8)	18.1 (IQR: 15.4–20.1)	<0.001
MAPSE (mm)	12.0 (IQR: 10.0–14.0)	14.0 (IQR: 12.0–16.0)	<0.001
RVEDD (mm)	34.0 (IQR: 30.0–38.0)	33.0 (IQR: 30.0–37.0)	0.31
LVEDD (mm)	55.0 (IQR: 50.0–62.3)	48.0 (IQR: 44.0–54.0)	<0.001
ePAPS	26.5 (IQR: 20.0–37.0)	20.5 (IQR: 17.3–24.8)	<0.001
Cardiac output (L/min)	4.60 ± 1.40	5.54 ± 1.40	<0.001
Aortic Vmax (m/s)	1.3 (IQR: 1.2–1.8)	1.2 (IQR: 1.1–1.4)	<0.001

LVEDD—left ventricular end-diastolic diameter, LVEF—left ventricular ejection fraction, LVEDV—left ventricular end-diastolic volume, LVESV—left ventricular end-systolic volume, LVESV—left ventricular end-systolic volume, E/A—peak velocity of blood flow during left ventricular relaxation in early diastole/peak velocity of flow in late diastole caused by atrial contraction, E/e′—left ventricular transmitral early diastolic filling velocity/left ventricular early diastolic myocardial velocity, E/E′ lateral-peak velocity of blood flow during left ventricular relaxation in early diastole/lateral left ventricular early diastolic myocardial velocity, E/E′ septal-left ventricular transmitral early diastolic filling velocity/septal wall of left ventricular early diastolic myocardial velocity, MV Dec T—mitral valve deceleration time, S′ lateral—systolic excursion velocity of the lateral wall of the left ventricle, S′ septal—systolic excursion velocity of the septum of the left ventricle, LAVI—left atrial volume index, MAPSE—mitral annular plane systolic excursion, N/A—not applicable.

**Table 3:** LVPs and TWA in patients with CHF and the control group.

	Chronic Heart Failure (*n* = 100)	Control (*n* = 50)	*p*-Value
LVPs (Yes/No)	Yes—34 (34%) No—66 (66%)	Yes—6 (12%) No—44 (88%)	<0.01
TWA (Yes/No)	Yes—21 (21%) No—79 (79%)	Yes—1 (2%) No—49 (98%)	<0.01

LVPs—late ventricular potentials, TWA—T-wave alternans, CHF—chronic heart failure.

**Table 4:** HRV parameters in patients with CHF and the control group.

	Chronic Heart Failure (*n* = 100)	Control (*n* = 50)	*p*-Value
SDNN (ms)	74.0 (IQR: 56.0–96.0)	105.0 (IQR: 74.3–132.0)	<0.001
SDANN (ms)	67.3 ± 27.0	81.0 ± 32.7	<0.01
SDNN Index (ms)	34.0 (IQR: (26.0–45.3)	48.0 (IQR: 35.3–63.8)	<0.001
RMSSD	23.0 (IQR: 16.0–34.5)	30.5 (IQR: 21.0–43.0)	0.03
PNN50 (%)	3.0 (IQR: 0.0–8.0)	5.0 (IQR: 2.0–11.0)	0.06
Triangular index (ms)	17.6 (IQR: 12.8–22.9)	27.0 (IQR: 16.4–34.5)	<0.001
VLF (Hz)	845.0 (IQR: 544.0–1591.0)	1450.0 (IQR: 680.0–2159.0)	0.02
LF (Hz)	179.0 (IQR: 92.2–359.0)	353.0 (IQR: 169.0–633.0)	<0.001
HF (Hz)	76.8 (IQR: 35.9–181.0)	104.0 (IQR: 58.5–241.0)	0.11
Deceleration capacity (ms)	3.8 (IQR: 2.4–5.6)	5.8 (IQR: 4.1–7.2)	<0.001
Acceleration capacity (ms)	−4.3 (IQR: −6.3–−2.9)	−6.5 (IQR:−7.8–−4.2)	<0.001

HRV—heart rate variability, CHF—chronic heart failure, SDNN—standard deviation of RR intervals for the entire duration, SDANN—standard deviation of the averages of NN intervals in each 5 min segment across the entire recording, SDNN index—mean of the 5 min normal-to-normal intervals throughout the complete recording, RMSSD—square root of the mean of the squares of the successive differences between adjacent NN intervals, PNN50—ratio of NN50 to the total count of NN intervals, vlF—very low frequency, lF—low frequency, hF—high frequency.

## Data Availability

The data presented in this study are available within the article.

## References

[1] Heusch G. (2024). Myocardial ischemia/reperfusion: Translational pathophysiology of ischemic heart disease. Med.

[2] Jensen R.V., Hjortbak M.V., Bøtker H.E. (2020). Ischemic Heart Disease: An Update. Semin. Nucl. Med..

[3] Severino P., D’Amato A., Pucci M., Infusino F., Adamo F., Birtolo L.I., Netti L., Montefusco G., Chimenti C., Lavalle C. (2020). Ischemic Heart Disease Pathophysiology Paradigms Overview: From Plaque Activation to Microvascular Dysfunction. Int. J. Mol. Sci..

[4] Khan M.A., Hashim M.J., Mustafa H., Baniyas M.Y., Al Suwaidi S.K.B.M., AlKatheeri R., Alblooshi F.M.K., Almatrooshi M.E.A.H., Alzaabi M.E.H., Al Darmaki R.S. (2020). Global Epidemiology of Ischemic Heart Disease: Results from the Global Burden of Disease Study. Cureus.

[5] Severino P., D’Amato A., Pucci M., Infusino F., Birtolo L.I., Mariani M.V., Lavalle C., Maestrini V., Mancone M., Fedele F. (2020). Ischemic Heart Disease and Heart Failure: Role of Coronary Ion Channels. Int. J. Mol. Sci..

[6] Pagliaro B.R., Cannata F., Stefanini G.G., Bolognese L. (2020). Myocardial Ischemia and Coronary Disease in Heart Failure. Heart Fail. Rev..

[7] Silverdal J., Sjöland H., Bollano E., Pivodic A., Dahlström U., Fu M. (2020). Prognostic Impact Over Time of Ischaemic Heart Disease vs. Non-Ischaemic Heart Disease in Heart Failure. ESC Heart Fail..

[8] Wang K., Tian J., Zheng C., Yang H., Ren J., Liu Y., Han Q., Zhang Y. (2021). Interpretable Prediction of 3-Year All-Cause Mortality in Patients with Heart Failure Caused by Coronary Heart Disease Based on Machine Learning and SHAP. Comput. Biol. Med..

[9] Tymińska A., Ozierański K., Balsam P., Maciejewski C., Wancerz A., Brociek E., Marchel M., Crespo-Leiro M.G., Maggioni A.P., Drożdż J. (2022). Ischemic Cardiomyopathy versus Non-Ischemic Dilated Cardiomyopathy in Patients with Reduced Ejection Fraction-Clinical Characteristics and Prognosis Depending on Heart Failure Etiology (Data from European Society of Cardiology Heart Failure Registries). Biology.

[10] Groenewegen A., Rutten F.H., Mosterd A., Hoes A.W. (2020). Epidemiology of Heart Failure. Eur. J. Heart Fail..

[11] Al-Zaiti S.S., Fallavollita J.A., Canty J.M., Carey M.G. (2014). Electrocardiographic Predictors of Sudden and Non-Sudden Cardiac Death in Patients with Ischemic Cardiomyopathy. Heart Lung.

[12] Truong D.T., Cuong H.X., Oanh N.O. (2021). The Relationship between NT-ProBNP and Clinical, Paraclinical Characteristics in Stable Ischemic Heart Disease Patients with Heart Failure. Intern. Med. J. Vietnam.

[13] Kinoshita T., Hashimoto K., Yoshioka K., Miwa Y., Yodogawa K., Watanabe E., Nakamura K., Nakagawa M., Nakamura K., Watanabe T. (2020). Risk Stratification for Cardiac Mortality Using Electrocardiographic Markers Based on 24-Hour Holter Recordings: The JANIES-SHD Study. J. Cardiol..

[14] Freund O., Caspi I., Alcalay I., Brezis M.R., Frydman S., Bornstein G. (2023). An Old Diagnostic Tool for New Indications: Inpatient Holter ECG for Conditions Other than Syncope or Stroke. Sci. Rep..

[15] Ksela J., Rupert L., Djordjevic A., Antonic M., Avbelj V., Jug B. (2022). Altered Heart Rate Turbulence and Variability Parameters Predict 1-Year Mortality in Heart Failure with Preserved Ejection Fraction. J. Cardiovasc. Dev. Dis..

[16] Nishimura T., Senoo K., Makino M., Munakata J., Tomura N., Shimoo S., Iwakoshi H., Shiraishi H., Matoba S. (2023). Prediction Model for the New Onset of Atrial Fibrillation Combining Features of 24-Hour Holter Electrocardiogram with 12-Lead Electrocardiogram. Int. J. Cardiol. Heart Vasc..

[17] Lauder L., Scholz S.S., Ewen S., Lettner C., Ukena C., Böhm M., Mahfoud F. (2020). Accuracy of Pulse Rate Derived from 24-h Ambulatory Blood Pressure Monitoring Compared with Heart Rate from 24-h Holter-ECG. J. Hypertens..

[18] Chua S.K., Chen L.C., Lien L.M., Lo H.M., Liao Z.Y., Chao S.P., Chuang C.Y., Chiu C.Z. (2020). Comparison of Arrhythmia Detection by 24-Hour Holter and 14-Day Continuous Electrocardiography Patch Monitoring. Acta Cardiol. Sin..

[19] Kwon S., Lee S.R., Choi E.K., Ahn H.J., Song H.S., Lee Y.S., Oh S. (2021). Validation of Adhesive Single-Lead ECG Device Compared with Holter Monitoring among Non-Atrial Fibrillation Patients. Sensors.

[20] Holkeri A., Eranti A., Haukilahti M.A.E., Kerola T., Kenttä T.V., Tikkanen J.T., Anttonen O., Noponen K., Seppänen T., Rissanen H. (2020). Predicting Sudden Cardiac Death in a General Population Using an Electrocardiographic Risk Score. Heart.

[21] Zorzi A., Vessella T., De Lazzari M., Cipriani A., Menegon V., Sarto G., Spagnol R., Merlo L., Pegoraro C., Marra M.P. (2020). Screening Young Athletes for Diseases at Risk of Sudden Cardiac Death: Role of Stress Testing for Ventricular Arrhythmias. Eur. J. Prev. Cardiol..

[22] Kwon J.M., Kim K.H., Jeon K.H., Lee S.Y., Park J., Oh B.H. (2020). Artificial Intelligence Algorithm for Predicting Cardiac Arrest Using Electrocardiography. Scand. J. Trauma Resusc. Emerg. Med..

[23] Wachter R., Gröschel K., Gelbrich G., Hamann G.F., Kermer P., Liman J., Seegers J., Wasser K., Schulte A., Jürries F. (2017). Holter-Electrocardiogram-Monitoring in Patients with Acute Ischaemic Stroke (Find-AFRANDOMISED): An Open-Label Randomised Controlled Trial. Lancet Neurol..

[24] Gladstone D.J., Dorian P., Spring M., Panzov V., Mamdani M., Healey J.S., Thorpe K.E., EMBRACE Steering Committee and Investigators (2015). Atrial Premature Beats Predict Atrial Fibrillation in Cryptogenic Stroke: Results from the EMBRACE Trial. Stroke.

[25] Hingorani P., Karnad D.R., Rohekar P., Kerkar V., Lokhandwala Y.Y., Kothari S. (2016). Arrhythmias Seen in Baseline 24-Hour Holter ECG Recordings in Healthy Normal Volunteers During Phase 1 Clinical Trials. J. Clin. Pharmacol..

[26] Kasahara K., Shiobara M., Nakamura S., Yamashiro K., Yana K., Ono T. Sudden Cardiac Arrest Risk Stratification Based on 24-Hour Holter ECG Statistics. Proceedings of the 2015 37th Annual International Conference of the IEEE Engineering in Medicine and Biology Society (EMBC).

[27] Cygankiewicz I., Zareba W., Vazquez R., Bayes-Genis A., Pascual D., Macaya C., Almendral J., Fiol M., Bardaji A., Gonzalez-Juanatey J.R. (2009). Risk Stratification of Mortality in Patients with Heart Failure and Left Ventricular Ejection Fraction >35%. Am. J. Cardiol..

[28] Xhyheri B., Manfrini O., Mazzolini M., Pizzi C., Bugiardini R. (2012). Heart Rate Variability Today. Prog. Cardiovasc. Dis..

[29] Sibrecht G., Piskorski J., Krauze T., Guzik P. (2023). Heart Rate Asymmetry, Its Compensation, and Heart Rate Variability in Healthy Adults during 48-h Holter ECG Recordings. J. Clin. Med..

[30] Bolanos M., Nazeran H., Haltiwanger E. (2006). Comparison of Heart Rate Variability Signal Features Derived from Electrocardiography and Photoplethysmography in Healthy Individuals. Conf. Proc. IEEE Eng. Med. Biol. Soc..

[31] Al-Zaiti S.S., Pietrasik G., Carey M.G., Alhamaydeh M., Canty J.M., Fallavollita J.A. (2019). The Role of Heart Rate Variability, Heart Rate Turbulence, and Deceleration Capacity in Predicting Cause-Specific Mortality in Chronic Heart Failure. J. Electrocardiol..

[32] Fang S.C., Wu Y.L., Tsai P.S. (2020). Heart Rate Variability and Risk of All-Cause Death and Cardiovascular Events in Patients with Cardiovascular Disease: A Meta-Analysis of Cohort Studies. Biol. Res. Nurs..

[33] Guzik P., Piskorski J., Barthel P., Bauer A., Müller A., Junk N., Ulm K., Malik M., Schmidt G. (2012). Heart Rate Deceleration Runs for Postinfarction Risk Prediction. J. Electrocardiol..

[34] You T., Luo C., Zhang K., Zhang H. (2021). Electrophysiological Mechanisms Underlying T-Wave Alternans and Their Role in Arrhythmogenesis. Front. Physiol..

[35] Quan X.Q., Zhou H.L., Ruan L., Lv J.G., Yao J.H., Yao F., Huang K., Zhang C.T. (2014). Ability of Ambulatory ECG-Based T-Wave Alternans to Modify Risk Assessment of Cardiac Events: A Systematic Review. BMC Cardiovasc. Disord..

[36] Nishibe T., Yamashiro K., Yana K., Ono T. T-Wave Alternans Search over 24 Hour Holter ECG Recordings Based on Singular Value Decomposition. Proceedings of the 2013 35th Annual International Conference of the IEEE Engineering in Medicine and Biology Society (EMBC).

[37] Lewek J., Ptaszynski P., Klingenheben T., Cygankiewicz I. (2017). The Clinical Value of T-Wave Alternans Derived from Holter Monitoring. Europace.

[38] Hashimoto K., Harada N. (2023). Recent Progress of Holter-Based Late Potential for Predicting Serious Cardiac Events and Its Implications and Future Challenges. J. Electrocardiol..

[39] Hashimoto K., Amino M., Yoshioka K., Kasamaki Y., Kinoshita T., Ikeda T. (2021). Combined Evaluation of Ambulatory-Based Late Potentials and Nonsustained Ventricular Tachycardia to Predict Arrhythmic Events in Patients with Previous Myocardial Infarction: A Japanese Noninvasive Electrocardiographic Risk Stratification of Sudden Cardiac Death (JANIES) Substudy. Ann. Noninvasive Electrocardiol..

[40] McDonagh T.A., Metra M., Adamo M., Gardner R.S., Baumbach A., Böhm M., Burri H., Butler J., Čelutkienė J., Chioncel O. (2021). 2021 ESC Guidelines for the Diagnosis and Treatment of Acute and Chronic Heart Failure. Eur. Heart J..

[41] Shaffer F., Ginsberg J.P. (2017). An Overview of Heart Rate Variability Metrics and Norms. Front. Public Health.

[42] Hu W., Jin X., Zhang P., Yu Q., Yin G., Lu Y., Xiao H., Chen Y., Zhang D. (2016). Deceleration and Acceleration Capacities of Heart Rate Associated with Heart Failure with High Discriminating Performance. Sci. Rep..

[43] Vozda M., Cerny M. (2015). Methods for Derivation of Orthogonal Leads from 12-Lead Electrocardiogram: A Review. Biomed. Signal Process. Control.

[44] Nademanee K., Veerakul G., Chandanamattha P., Chaothawee L., Ariyachaipanich A., Jirasirirojanakorn K., Likittanasombat K., Bhuripanyo K., Ngarmukos T. (2011). Prevention of ventricular fibrillation episodes in Brugada syndrome by catheter ablation over the anterior right ventricular outflow tract epicardium. Circulation.

[45] Matsuzaki A., Yoshioka K., Amino M., Shima M., Hashida T., Fujibayashi D., Kanda S., Kobayashi Y., Tanabe T., Ikari Y. (2014). Usefulness of Continuous 24-Hour Ventricular Late Potential to Predict Prognosis in Patients with Heart Failure. Tokai J. Exp. Clin. Med..

[46] Pepine C.J., Nichols W.W. (2007). The pathophysiology of chronic ischemic heart disease. Clin. Cardiol..

[47] El-Sherif N., Boutjdir M., Turitto G. (2017). Sudden Cardiac Death in Ischemic Heart Disease: Pathophysiology and Risk Stratification. Card. Electrophysiol. Clin..

[48] Maas A.H., Appelman Y.E. (2010). Gender differences in coronary heart disease. Neth. Heart J..

[49] Hansen S., Rasmussen V., Torp-Pedersen C., Jensen G.B. (2008). QT intervals and QT dispersion determined from a 12-lead 24-hour Holter recording in patients with coronary artery disease and patients with heart failure. Ann. Noninvasive Electrocardiol..

[50] Liew R. (2011). Electrocardiogram-based predictors of sudden cardiac death in patients with coronary artery disease. Clin. Cardiol..

[51] La Rovere M.T., Pinna G.D., Maestri R., Mortara A., Capomolla S., Febo O., Ferrari R., Franchini M., Gnemmi M., Opasich C. (2003). Short-term heart rate variability strongly predicts sudden cardiac death in chronic heart failure patients. Circulation.

[52] Chen W., Zheng L., Li K., Wang Q., Liu G., Jiang Q. (2016). A Novel and Effective Method for Congestive Heart Failure Detection and Quantification Using Dynamic Heart Rate Variability Measurement. PLoS ONE.

[53] Schneider A., Hampel R., Ibald-Mulli A., Zareba W., Schmidt G., Schneider R., Rückerl R., Couderc J.P., Mykins B., Oberdörster G. (2010). Changes in deceleration capacity of heart rate and heart rate variability induced by ambient air pollution in individuals with coronary artery disease. Part. Fibre Toxicol..

[54] Ricca-Mallada R., Migliaro E.R., Piskorski J., Guzik P. (2012). Exercise training slows down heart rate and improves deceleration and acceleration capacity in patients with heart failure. J. Electrocardiol..

[55] Yan L., Jin J., Zhao X., Huang X., Zhu W., Jiang S., Gao M., Yuan J. (2020). Heart rate acceleration and deceleration capacities associated with circadian blood pressure variation. Ann. Noninvasive Electrocardiol..

[56] Hautala A.J., Karjalainen J., Kiviniemi A.M., Kinnunen H., Mäkikallio T.H., Huikuri H.V., Tulppo M.P. (2010). Physical activity and heart rate variability measured simultaneously during waking hours. Am. J. Physiol. Heart Circ. Physiol..

[57] Lombardi F., Stein P.K. (2011). Origin of heart rate variability and turbulence: An appraisal of autonomic modulation of cardiovascular function. Front. Physiol..

[58] Fogt D.L., Cooper P.J., Freeman C.N., Kalns J.E., Cooke W.H. (2009). Heart rate variability to assess combat readiness. Mil. Med..

[59] Liu H., Zhan P., Shi J., Wang G., Wang B., Wang W. (2018). A refined method of quantifying deceleration capacity index for heart rate variability analysis. Biomed. Eng. Online.

[60] d’Unienville N.M.A., Nelson M.J., Bellenger C.R., Blake H.T., Buckley J.D. (2022). Heart-Rate Acceleration Is Linearly Related to Anaerobic Exercise Performance. Int. J. Sports Physiol. Perform..

[61] Schneider C., Wiewelhove T., Raeder C., Flatt A.A., Hoos O., Hottenrott L., Schumbera O., Kellmann M., Meyer T., Pfeiffer M. (2019). Heart Rate Variability Monitoring During Strength and High-Intensity Interval Training Overload Microcycles. Front. Physiol..

[62] Arsenos P., Manis G., Gatzoulis K.A., Dilaveris P., Gialernios T., Angelis A., Papadopoulos A., Venieri E., Trikas A., Tousoulis D. (2016). Deceleration Capacity of Heart Rate Predicts Arrhythmic and Total Mortality in Heart Failure Patients. Ann. Noninvasive Electrocardiol..

[63] Giunta S., Xia S., Pelliccioni G., Olivieri F. (2024). Autonomic nervous system imbalance during aging contributes to impair endogenous anti-inflammaging strategies. Geroscience.

[64] Duan S., Wang J., Yu F., Song L., Liu C., Sun J., Deng Q., Wang Y., Zhou Z., Guo F. (2022). Enrichment of the Postdischarge GRACE Score with Deceleration Capacity Enhances the Prediction Accuracy of the Long-Term Prognosis after Acute Coronary Syndrome. Front. Cardiovasc. Med..

[65] Li Z.W., Zhao H.M., Wang J. (2021). Metabolism and Chronic Inflammation: The Links between Chronic Heart Failure and Comorbidities. Front. Cardiovasc. Med..

[66] Khan M.S., Samman Tahhan A., Vaduganathan M., Greene S.J., Alrohaibani A., Anker S.D., Vardeny O., Fonarow G.C., Butler J. (2020). Trends in prevalence of comorbidities in heart failure clinical trials. Eur. J. Heart Fail..

[67] Triposkiadis F.K., Skoularigis J. (2012). Prevalence and importance of comorbidities in patients with heart failure. Curr. Heart Fail. Rep..

[68] Taçoy G., Açikgöz K., Kocaman S.A., Ozdemir M., Cengel A. (2010). Is there a relationship between obesity, heart rate variability, and inflammatory parameters in heart failure?. J. Cardiovasc. Med..

[69] Levey A.S., Eckardt K.U., Tsukamoto Y., Levin A., Coresh J., Rossert J., De Zeeuw D., Hostetter T.H., Lameire N., Eknoyan G. (2005). Definition and classification of chronic kidney disease: A position statement from Kidney Disease: Improving Global Outcomes (KDIGO). Kidney Int..

[70] Yadav R.L., Yadav P.K., Yadav L.K., Agrawal K., Sah S.K., Islam M.N. (2017). Association between obesity and heart rate variability indices: An intuition toward cardiac autonomic alteration—A risk of CVD. Diabetes Metab. Syndr. Obes..

[71] Correale M., Paolillo S., Mercurio V., Ruocco G., Tocchetti C.G., Palazzuoli A. (2021). Non-cardiovascular comorbidities in heart failure patients and their impact on prognosis. Kardiol. Pol..

[72] Ren L., Fang X., Wang Y., Qi G. (2011). T-wave alternans and heart rate variability: A comparison in patients with myocardial infarction with or without diabetes mellitus. Ann. Noninvasive Electrocardiol..

[73] Seferović P.M., Petrie M.C., Filippatos G.S., Anker S.D., Rosano G., Bauersachs J., Paulus W.J., Komajda M., Cosentino F., de Boer R.A. (2018). Type 2 diabetes mellitus and heart failure: A position statement from the Heart Failure Association of the European Society of Cardiology. Eur. J. Heart Fail..

[74] Riaz B., Khan M.A., Ali H., Majeed S.M.I. (2018). Correlation of Signal Averaged ECG Parameters with Left Ventricular Mass Index in Patients with Systemic Arterial Hypertension. Pak. J. Physiol..

[75] Stoyell-Conti F.F., Santos F., Machi J.F., Hernandez D.R., Barboza C.A., Irigoyen M.C., De Angelis K., Morris M. (2018). Measurement of Mouse Heart Rate Variability using Echocardiographic System. J. Cardiovasc. Echogr..

[76] Petelczyc M., Zebrowski J.J., Baranowski R., Chojnowska L. (2010). Stochastic analysis of heart rate variability and its relation to echocardiography parameters in hypertrophic cardiomyopathy patients. Physiol. Meas..

[77] Mele D., Andrade A., Bettencourt P., Moura B., Pestelli G., Ferrari R. (2020). From left ventricular ejection fraction to cardiac hemodynamics: Role of echocardiography in evaluating patients with heart failure. Heart Fail. Rev..

[78] Pastore M.C., Mandoli G.E., Aboumarie H.S., Santoro C., Bandera F., D’Andrea A., Benfari G., Esposito R., Evola V., Sorrentino R. (2020). Basic and advanced echocardiography in advanced heart failure: An overview. Heart Fail. Rev..

[79] Arora R., Krummerman A., Vijayaraman P., Rosengarten M., Suryadevara V., Lejemtel T., Ferrick K.J. (2004). Heart rate variability and diastolic heart failure. Pacing Clin. Electrophysiol..

[80] Monasterio V., Laguna P., Cygankiewicz I., Vázquez R., Bayés-Genís A., de Luna A.B., Martínez J.P. (2012). Average T-wave alternans activity in ambulatory ECG records predicts sudden cardiac death in patients with chronic heart failure. Heart Rhythm.

[81] Santangeli P., Infusino F., Sgueglia G.A., Sestito A., Lanza G.A. (2008). Ventricular late potentials: A critical overview and current applications. J. Electrocardiol..

[82] Perkiömäki J., Exner D.V., Piira O.P., Kavanagh K., Lepojärvi S., Talajic M., Karvonen J., Philippon F., Junttila J., Coutu B. (2015). Heart Rate Turbulence and T-Wave Alternans in Patients with Coronary Artery Disease: The Influence of Diabetes. Ann. Noninvasive Electrocardiol..

[83] Lutfi M.F. (2017). Ventricular late potential in cardiac syndrome X compared to coronary artery disease. BMC Cardiovasc. Disord..

[84] Bangalore S., Sawhney S., Messerli F.H. (2008). Relation of beta-blocker-induced heart rate lowering and cardioprotection in hypertension. J. Am. Coll. Cardiol..

[85] Goupil R., Dupuis D., Troyanov S., Madore F., Agharazii M. (2016). Heart rate dependent and independent effects of beta-blockers on central hemodynamic parameters: A propensity score analysis. J. Hypertens..

[86] Elghozi J.L., Julien C. (2007). Sympathetic control of short-term heart rate variability and its pharmacological modulation. Fundam. Clin. Pharmacol..

[87] Muthalaly R.G., Evans R.M. (2020). Applications of Machine Learning in Cardiac Electrophysiology. Arrhythm. Electrophysiol. Rev..

[88] Agliari E., Barra A., Barra O.A., Fachechi A., Franceschi Vento L., Moretti L. (2020). Detecting cardiac pathologies via machine learning on heart-rate variability time series and related markers. Sci. Rep..

[89] Ishaque S., Khan N., Krishnan S. (2021). Trends in Heart-Rate Variability Signal Analysis. Front. Digit. Health.

[90] Freund O., Caspi I., Shacham Y., Frydman S., Biran R., Abu Katash H., Zornitzki L., Bornstein G. (2022). Holter ECG for Syncope Evaluation in the Internal Medicine Department—Choosing the Right Patients. J Clin Med..

[91] Uppoor R.B., Patel K. (2022). Syncope: Diagnostic Yield of Various Clinical Investigations. Cureus..

[92] Canepa M., Fonseca C., Chioncel O., Laroche C., Crespo-Leiro M.G., Coats A.J.S., Mebazaa A., Piepoli M.F., Tavazzi L., Maggioni A.P. (2018). Performance of Prognostic Risk Scores in Chronic Heart Failure Patients Enrolled in the European Society of Cardiology Heart Failure Long-Term Registry. JACC Heart Fail..

[93] Boehmer J.P., Hariharan R., Devecchi F.G., Smith A.L., Molon G., Capucci A., An Q., Averina V., Stolen C.M., Thakur P.H. (2017). A Multisensor Algorithm Predicts Heart Failure Events in Patients with Implanted Devices: Results from the MultiSENSE Study. JACC Heart Fail..

[94] D’Amato A., Severino P., Mancone M., Mariani M.V., Prosperi S., Colombo L., Myftari V., Cestiè C., Labbro Francia A., Germanò R. (2024). Prognostic Assessment of HLM Score in Heart Failure Due to Ischemic Heart Disease: A Pilot Study. J. Clin. Med..

[95] Levy W.C., Mozaffarian D., Linker D.T., Sutradhar S.C., Anker S.D., Cropp A.B., Anand I., Maggioni A., Burton P., Sullivan M.D. (2006). The Seattle Heart Failure Model: Prediction of Survival in Heart Failure. Circulation.

